# From Battlefield to Blood Bank: Leveraging the Whole Blood Resurgence to Advance Global Health Equity

**DOI:** 10.1002/wjs.70175

**Published:** 2025-11-20

**Authors:** Ana M. Reyes, Stephen Q. Conrad, Talia R. Arcieri, Jonathan P. Meizoso, Julie Y. Valenzuela

**Affiliations:** ^1^ Division of Trauma Surgical Critical Care and Burns Dewitt Daughtry Family Department of Surgery University of Miami Miami Florida USA; ^2^ University of Miami Miller School of Medicine Miami Florida USA

## 1 From Component Therapy to Whole Blood Revival

In trauma care, a century‐old practice is making a comeback: transfusion of whole blood. During the First and Second World Wars, whole blood was the standard transfusion product for treating combat casualties. But as technology evolved, advances in fractionation made it possible to separate whole blood into units of red blood cells, plasma, and platelets. The efficiency of treating multiple patients from a single donation and growing concerns about hepatitis risk with whole blood prompted the medical field to advocate for transfusion of blood components. And by the 1970s, component therapy had become the global standard for hemorrhage resuscitation. Recent military conflicts, however, have reignited interest in whole blood. In conflicts in Iraq and Afghanistan, the challenge of maintaining components in austere environments prompted the use of fresh whole blood from “walking blood banks”, or pre‐screened donor soldiers ready to give blood when needed. Data from these conflicts emerged suggesting that fresh whole blood led to equal or even improved survival compared to component therapy. Civilian trauma centers soon took notice, and a recent meta‐analysis found that stored low‐titer O whole blood improved 24‐h survival and reduced late mortality in both adults and children [1].

## 2 Blood System Challenges in LMICs

Although high‐income countries are now rediscovering whole blood as an innovation, much of the world never left it behind. In many low‐ and middle‐income countries (LMICs), whole blood has remained the primary transfusion product due to the immense infrastructure required for traditional blood banking: centrifugation and fractionation equipment, skilled laboratory personnel, continuous cold‐chain storage, expensive reagents for infectious disease testing, and centralized data systems for donor tracking and inventory management. Consequently, traditional blood banking is often limited to major urban centers and in some low‐income countries, even the largest blood banks do not perform component separation. Blood shortages remain staggering, estimated at more than 100 million units globally each year, with deficits in every country in sub‐Saharan Africa, south Asia, and Oceania [2]. And women and children bear the brunt of this scarcity. In sub‐Saharan Africa, nearly one third of maternal deaths from hemorrhage are attributable to lack of blood availability and untreated anemia still accounts for a large share of childhood deaths [3]. Challenges include both inadequate donation rates and insufficient infrastructure to support modern blood banking. In many hospitals in Latin America, south Asia, and Africa, hospital‐based blood banks rely on family or replacement donors, and even other hospitalized patients, as donors to generate their blood supply.

## 3 Shared Lessons for a Global Problem

Yet the global blood ecosystem is fragile across many contexts—not only in LMICs. Even in high‐income countries, “blood deserts” are emerging in rural regions, disaster zones, and resource‐constrained hospitals. Global crises, from pandemics to climate‐related disasters have exposed how blood supply chains can rapidly collapse under pressure. Moreover, inequities persist even where blood is technically available, as women have been shown to receive transfusions less frequently or later in resuscitation [4]. The renewed interest in whole blood, therefore, represents an opportunity to address long‐standing inequities in access to safe, timely blood transfusion across a variety of settings. But this moment should not be limited to advancing care for patients in wealthy nations. Rather, it should catalyze reciprocal learning and foster a shared platform for mutual advancement to strengthen blood systems globally.

## 4 Optimizing Efficiency Across Contexts

Scaling up of whole blood programs brings opportunities for shared learning in logistics, efficiency, and waste reduction. Cost savings is one area of potential. In the United States, one hospital found that using whole blood for trauma resuscitation saved $3.4 million compared to component therapy by reducing overall blood products used and wasted [5]. In Zimbabwe, producing one unit of whole blood was 10% cheaper than producing one unit of packed red blood cells [6]. Whole blood may offer a more financially sustainable and operationally efficient approach over component therapy when appropriate. Additionally, as whole blood programs expand, technology to support inventory management must evolve. Emerging tools such as real‐time dashboards, predictive algorithms to anticipate demand, and meticulously planned redistribution networks for units nearing expiration offer promise for improving blood system efficiency and reducing waste. Greater investment in these tools, coupled with shared learning across contexts, could enable both low‐ and high‐resource systems to strengthen blood supply management.

## 5 Drones and Modern Transport Logistics

Expanding the reach of whole blood will also depend on overcoming barriers related to transport. Unmanned aerial vehicles (UAVs), or drones, are already rewriting the rules of blood transport logistics. Rwanda's drone delivery program, launched in 2017, revolutionized its blood distribution system by reducing delivery times from hours to minutes, cutting blood product waste, and enabling consistent access to blood even in remote, rural hospitals [7]. Similar programs in Ghana demonstrate that these programs are feasible, scalable, and warrant broader global investment [8]. In several high‐income countries, the use of drones for interhospital blood transport, pre‐hospital blood supply, and direct delivery to trauma scenes is beginning to be explored. Whether transporting blood over mountain ranges or bypassing urban congestion, drone delivery holds potential to create faster and more reliable blood distribution systems across both rural and urban settings worldwide.

## 6 Community Solutions for Blood System Resilience

In true blood deserts where capacity to store or receive blood is critically limited or non‐existent, community walking blood banks (WBBs) have been proposed as a pragmatic solution. The concept is not new: WBBs, first used during World War I, rely on a pool of pre‐screened donors who can be mobilized within minutes, providing fresh whole blood for emergency transfusions without the need for cold storage. At a hospital in northwestern Kenya, for instance, a community WBB enabled timely transfusions for patients with hemorrhage, with transfusion‐transmitted infection rates comparable to national standards for stored blood [9]. Similar programs have been proposed in other rural regions in LMICs where building conventional blood banks is prohibitively expensive. Importantly, high‐income countries are now adapting the same model for disaster readiness. For example in Southwest Texas, following mass‐casualty incidents in El Paso and Uvalde, a civilian WBB initiative called “Heroes in Arms” was created to maintain a registry of pre‐screened donors ready to respond during massive transfusion events [10]. These examples demonstrate that WBBs are not just an emergency improvisation, but they represent a scalable model that can strengthen blood system capacity in a variety of environments. A next step is rigorous evaluation to identify barriers and facilitators to WBB success, with key priorities including community engagement and trust, understanding donor motivation and cultural acceptance, and training healthcare workers in WBB protocols.

## 7 Bridging Innovation and Equity

The resurgence of whole blood represents more than a clinical innovation—it highlights a pivotal opportunity for global collaboration given that blood systems across contexts, from shortages in LMICs to rural areas of high‐income countries, face common challenges. As transfusion science and technology continue to advance, it is essential to ensure that regions where whole blood has long been the foundation of care are not left behind but are positioned to benefit fully from these innovations. The scientific community bears a collective responsibility to bridge innovation and equity so that safe, timely transfusion becomes a universal standard of care.

## Funding

The authors have nothing to report.

## Conflicts of Interest

The authors declare no conflicts of interest.

## References

[1] K. M. Morgan , E. A. Khalil , E. V. Feeney , et al., “The Efficacy of Low‐Titer Group O Whole Blood Compared With Component Therapy in Civilian Trauma Patients: A Meta‐Analysis,” Critical Care Medicine 52, no. 7 (July 2024): e390–e3404. 10.1097/CCM.0000000000006244. 38483205

[2] N. Roberts , S. James , M. Delaney , and C. Fitzmaurice , “The Global Need and Availability of Blood Products: A Modelling Study,” Lancet Haematology 6, no. 12 (December 2019): e606–e615, 10.1016/s2352-3026(19)30200-5. 31631023

[3] I. Bates , G. K. Chapotera , A. McKew , and N. van den Broek , “Maternal Mortality in Sub‐Saharan Africa: The Contribution of Ineffective Blood Transfusion Services,” BJOG 115, no. 11 (October 2008): 1331–1339, 10.1111/j.1471-0528.2008.01866.x. 18823485

[4] S. Clayton , M. Tremoglie‐Barkowski , C. M. Leeper , L. Lu , J. Brown , and P. C. Spinella , “Sex‐Based Disparities in Low‐Titer O Whole Blood Utilization and Mortality Among Severely Injured Trauma Patients,” supplement, Transfusion 65, no. S1 (May 2025): S156–S165, 10.1111/trf.18240. 40208115 PMC12035985

[5] R. C. Murphy , T. W. Johnson , T. J. Mack , et al., “Cost Savings of Whole Blood Versus Component Therapy at a Community Level 1 Trauma Center,” American Surgeon 90 (September 2024): 2156–2159, 10.1177/00031348241241712. 38591174

[6] N. Mafirakureva , H. Nyoni , S. Z. Nkomo , et al., “The Costs of Producing a Unit of Blood in Zimbabwe,” Transfusion 56, no. 3 (March 2016): 628–636, 10.1111/trf.13405. 26598799

[7] M. P. Nisingizwe , P. Ndishimye , K. Swaibu , et al., “Effect of Unmanned Aerial Vehicle (Drone) Delivery on Blood Product Delivery Time and Wastage in Rwanda: A Retrospective, Cross‐Sectional Study and Time Series Analysis,” Lancet Global Health 10, no. 4 (April 2022): e564–e569, 10.1016/s2214-109x(22)00048-1. 35303465

[8] M. Delaney , S. Telke , S. Zou , M. J. Williams , J. O. Aridi , and K. E. Rudd , “The BLOODSAFE Program: Building the Future of Access to Safe Blood in Sub‐Saharan Africa,” Transfusion 62, no. 11 (2022): 2282–2290, 10.1111/trf.17091. 36173295 PMC9643608

[9] P. Muiruri , “Kenya’s ‘Blood Desert’: Can Walking Donor Banks and Drones Help More Patients Survive?,” Guardian (2024), https://www.theguardian.com/global‐development/2024/apr/19/kenyas‐blood‐desert‐can‐walking‐donor‐banks‐and‐drones‐help‐more‐patients‐survive.

[10] E. P. Brigmon , J. Cirone , K. Harrell , et al., “Walking Blood Bank: A Plan to Ensure Self‐Sufficiency in an Era of Blood Shortage,” supplement, Trauma Surgery & Acute Care Open 9, no. S1 (January 2024): e001151, 10.1136/tsaco-2023-001151. 38196930 PMC10773437

